# Oxidative Stress Mediates the Disruption of Airway Epithelial Tight Junctions through a TRPM2-PLCγ1-PKCα Signaling Pathway

**DOI:** 10.3390/ijms14059475

**Published:** 2013-04-29

**Authors:** Rui Xu, Qi Li, Xiang-Dong Zhou, Juliy M. Perelman, Victor P. Kolosov

**Affiliations:** 1 Department of Respiratory Medicine, the Second Affiliated Hospital, Chongqing University of Medical Science, Chongqing 400010, China; E-Mails: chongyixurui@163.com (R.X.); lqlq198210@sina.com (Q.L.); 2 Far Eastern Scientific Center of Physiology and Pathology of Respiration, Russian Academy of Medical Sciences, Blagoveschensk 675000, Russia; E-Mails: cfpd@amur.ru (J.M.P.); kolosov@amur.ru (V.P.K.)

**Keywords:** oxidative stress, tight junctions, TRPM2, PLCγ1, PKCα

## Abstract

Oxidative stress has been implicated as an important contributing factor in the pathogenesis of several pulmonary inflammatory diseases. Previous studies have indicated a relationship between oxidative stress and the attenuation of epithelial tight junctions (TJs). In Human Bronchial Epithelial-16 cells (16HBE), we demonstrated the degradation of zonula occludens-1 (ZO-1), and claudin-2 exhibited a great dependence on the activation of the transient receptor potential melastatin (TRPM) 2 channel, phospholipase Cγ1 (PLCγ1) and the protein kinase Cα (PKCα) signaling cascade.

## 1. Introduction

Oxidative stress and free radical generation have been implicated as important contributing factors in the pathogenesis of acute exacerbation of chronic obstructive pulmonary disease (AECOPD). Reactive oxidant species (ROS) may indirectly cause the upregulation of histone acetyltransferase (HAT) activity in respiratory epithelial cells, leading to major inflammatory gene transcription [1]. The respiratory epithelial barrier acts as the first protective defense against allergens, microorganisms and particulate matter. Tight junctions (TJs) contribute as the major barrier components in epithelial monolayers, maintaining the apical-basolateral cell polarity and the integrity of the airway epithelial barrier [2]. Recent evidence suggests that TJs also participate in signal transduction mechanisms in epithelial cells [3].

It is useful to divide the TJ proteins into two separate categories: integral membrane proteins and peripheral membrane proteins. The integral membrane proteins include occludins, claudins and junctional adhesion molecules (JAMs). The peripheral membrane proteins include the scaffold PDZ-expression proteins, zonula occludens, (ZO)-1, ZO-2 and ZO-3. Of these TJ protein families, claudins are considered to be the most important components of the TJs at the interface of the basolateral and apical membranes of polarized epithelial cells. They determine the barrier properties of the cell-cell contact between two neighboring epithelial cells and regulate the paracellular permeability. Of the claudin family members, claudin-3, claudin-4 and claudin-5 were detected in rat type II alveolar epithelial cells [4,5]. Additionally, claudin-2 is also expressed in the human lung cell line A549 [6]. Compared with claudin-3, claudin-4 and claudin-5, claudin-2 is uniquely susceptible to H_2_O_2_ [7]. ZO-1, located between occludin and cytoskeletal proteins, was thought to affect paracellular permeability [8]. Previous studies have revealed an indispensable function for ZO-1 in the epithelial barrier in cornea [9], intestinal [10], brain [11] and airway cells [12]. Tight junction permeability is regulated through a variety of mechanisms, the most common of which involves the modulation of protein kinase C (PKC), particularly the alpha subtype [13,14]. However, the upstream pathway of PKCα induced by oxidative stress in airway epithelial injury has not been illustrated.

There is sufficient evidence that oxidative stress stimulates the formation of ADP-ribose and activates transient receptor potential melastatin (TRPM) 2 channel [15], which is responsible for the increase in cytosolic Ca^2+^ concentration [16]. Based on evidence demonstrating that the activation of PKCα is dependent upon the alternative phosphatidylinositol 4,5-bisphosphate (PIP2)-dependent pathway [17] and phospholipase Cγ1 (PLCγ1) is the main PLC subtype sensitive to hypoxia and oxidative stress [18,19], we hypothesized that under conditions of oxidative stress, the TRPM2-Ca^2+^-PLCγ1-PKCα cascade signaling pathway is responsible for the decreased expression of TJs and increased permeability in the airway epithelium.

## 2. Results and Discussion

### 2.1. H_2_O_2_ Exposure Activates PLCγ1 and Subsequently, PKCα in a TRPM2 Dependent Manner

**Figure 1:**
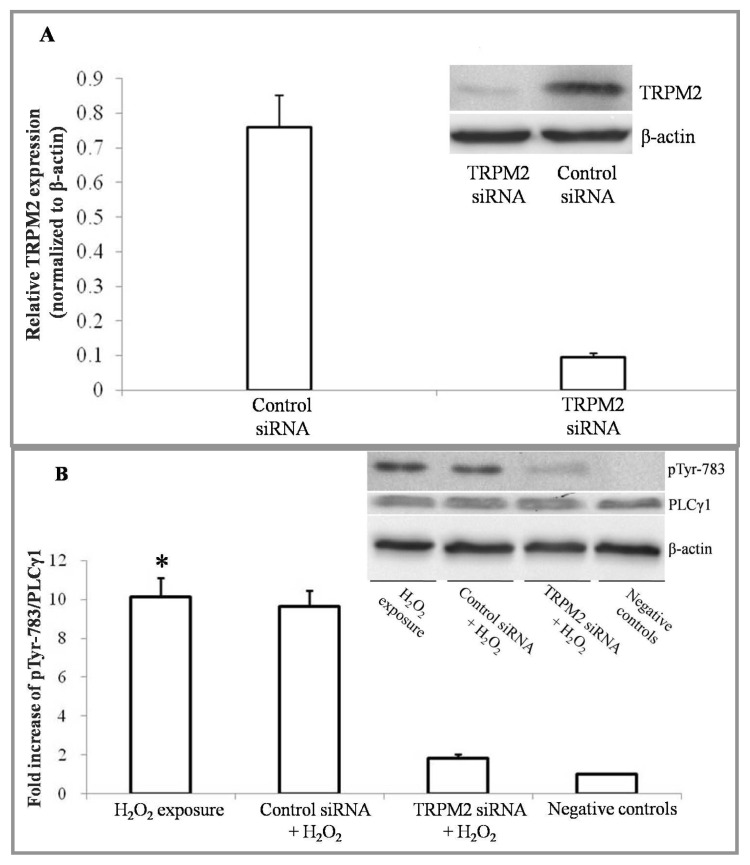

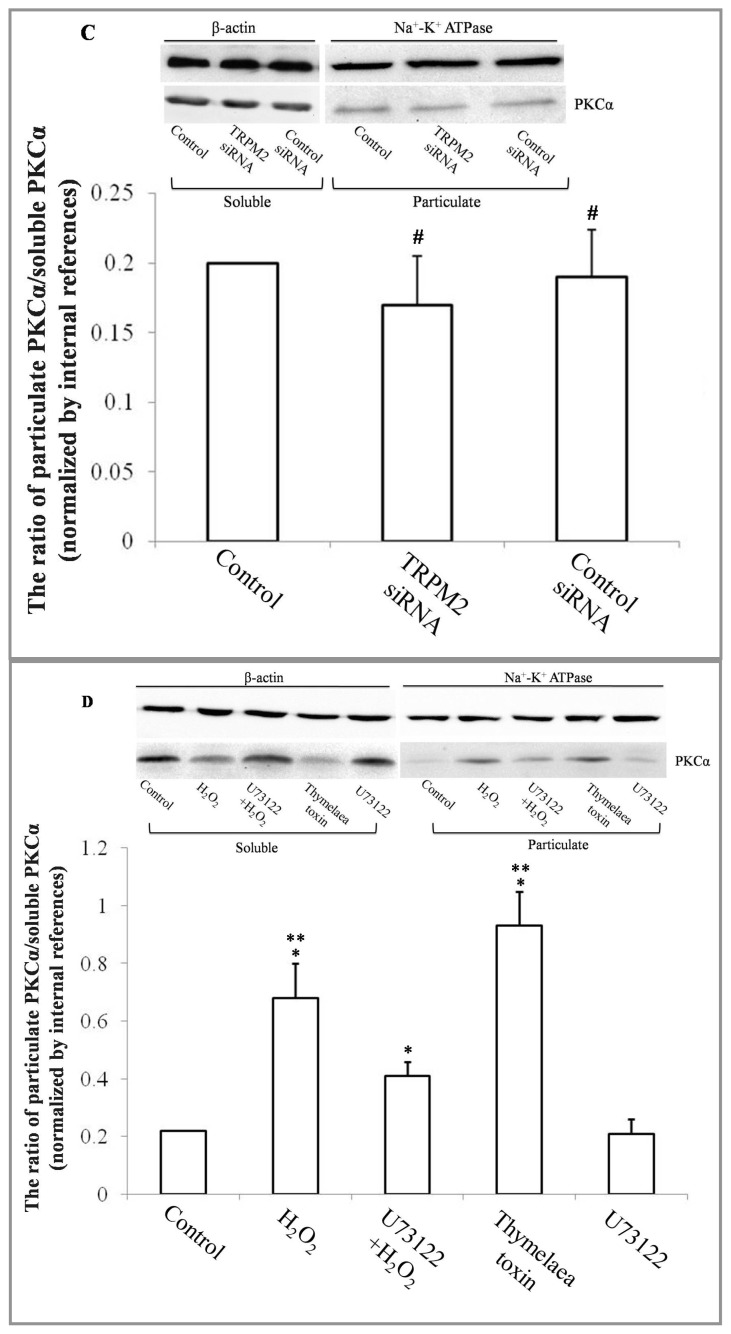
The activity of PLCγ1 and PKCα, estimated by Western blot analysis. (**A**) Compared to a control siRNA transfection, transient receptor potential melastatin (TRPM)-2 expression levels were reduced by >80% in the presence of a specific TRPM2 siRNA; (**B**) PLCγ1 and phosphorylated PLCγ1 at tyrosine 783 were detected by Western blot analysis. The protein levels were normalized with respect to β-actin. 16HBE cells treated with H_2_O_2_ free DMEM for 4 h were set as negative control. (*n* = 6 for each condition) * *p* < 0.05 for the TRPM2 siRNA transfection + H_2_O_2_ exposure group and negative controls; (**C**) PKCα was detected in both particulate and soluble extracts. To investigate whether TRPM2 depletion would influence the activity of PKCα, TRPM2 specific siRNA or control siRNA was transfected into 16HBE cells. (*n* = 6 for each condition), ^#^*p* > 0.05 compared to the control; (**D**) PKCα was detected in both particulate and soluble extracts. 16HBE cells treated with H_2_O_2_-free DMEM for 4 h were set as negative control, (*n* = 6 for each condition), * *p* < 0.05, compared to the negative control, ** *p* < 0.05, compared to either the U73122 + H_2_O_2_ group or the negative control.

It has been demonstrated that PLCγ1, highly expressed in lung tissue, is activated by ROS in several types of cells [18,19]. Studies of endothelial hyperpermeability have shown that H_2_O_2_ induced Ca^2+^ entry by the TRPM2 channel [15] and that PKCα activity was linked to the function of epithelial TJs [20]. Because the membrane distribution of activated PKCα is correlated with PIP2 localization [21], we hypothesized that the oxidative reaction increases the activation of PKCα through a TRPM2-Ca^2+^-PLCγ1 signaling pathway. PLCγ1 could be phosphorylated at Tyr771, 783 and 1245. However, phosphorylation by Syk at Tyr783 activated the enzymatic activity of PLCγ1 [22,23]. Therefore, the activity of PLCγ1 was assessed by examining the phosphorylation of PLCγ1 at tyrosine 783 using Western blot analysis. First, we demonstrated that transfection of 16HBE cells with TRPM2 small interfering RNA (siRNA) markedly and specifically diminished TRPM2 expression. The protein expression level of TRPM2 was successfully knocked down by >80% upon delivery of a specific siRNA (Figure 1A).

Following exposure to 0.5 mM H_2_O_2_ for 4 h, the phosphorylation level of PLCγ1 in 16HBE cells was significantly higher compared to those of TRPM2 deficient cells and negative controls (Figure 1B). The activity of PKCα was estimated by comparing the quantities of PKCα protein level in the particulate and soluble extracts (see Experimental Section). Thymelaea toxin (100 nM) pretreatments served as positive controls for PKCα activation. Before exposure to H_2_O_2_, the activity test for PKCα was performed to make sure whether TRPM2 depletion would cause an influence on the activity of PKCα. Compared to the negative control and control siRNA transfected group, TRPM2 depletion brought no significant changes on the activity of PKCα before H_2_O_2_ exposure (Figure 1C). After exposure to H_2_O_2_, the activity of PKCα exhibited an approximate three-fold increase compared to the negative control. However, in 16HBE cells pretreated with PLCγ inhibitor U73122 [24] (400 ng/mL), PKCα exhibited a poorer reaction to H_2_O_2_ (Figure 1D).

### 2.2. TRPM2 siRNA and Pretreatment with a PLCγ- or a PKCα-Specific Inhibitor Attenuate the Hyperpermeability Induced by an Oxidative Reaction

**Figure 2:**
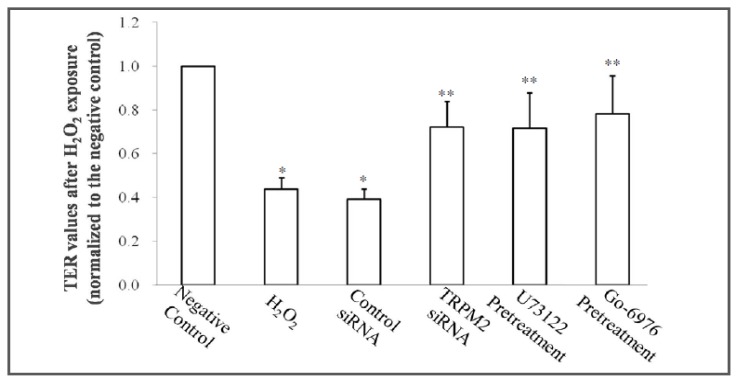
Effect of exogenous H_2_O_2_ individually or combined with TRPM2 siRNA, U73122 or Go-6976 on TER in 16HBE cells. TER values of each group after exposure to H_2_O_2_, were normalized to the average value of negative control after treatment with H_2_O_2_ free DMEM for 4 h, (*n* = 6 for each condition) * *p* < 0.05 compared to controls, ** *p* < 0.05 compared to the H_2_O_2_ group.

Epithelial barrier function was assessed by transepithelial electrical resistance (TER), as described in the experimental section. 16HBE cells exposed to H_2_O_2_ free DMEM culture medium were set as negative control. Following exposure to 0.5 mM H_2_O_2_ for 4 h, the TER values were recorded and normalized to the values of negative control. The TER values in 16HBE cells without any pretreatments decreased at approximately 56.2% after H_2_O_2_ stimulation. To address whether TRPM2 siRNA transfection or pretreatment with either a PLCγ1- or a PKCα-specific inhibitor could prevent the hyperpermeability induced by H_2_O_2_ exposure, 16HBE cells were pretreated with TRPM2 siRNA, U73122 (400 ng/mL) or Go-6976 (5 μM), respectively, before exposure to H_2_O_2_. Pretreatment of PLCγ or PKCα inhibitor brought no significant difference in TER before H_2_O_2_ exposure (Table 1). We estimated this was because a cytoplasm location of inactivated PKCα before positive treatment of PKCα activator, as previous studies indicated [14]. Compared to the 56.2% decrease in TER of non-pretreatment 16HBE cells, TRPM2-specific siRNA transfection, U73122 or Go-6976 pretreatment exhibited a significant attenuation in TER depletion after oxidative stress (Table 1, Figure 2).

### 2.3. Effect of TRPM2-PLCγ1-PKCα on ZO-1 and Claudin-2 Expression

**Figure 3:**
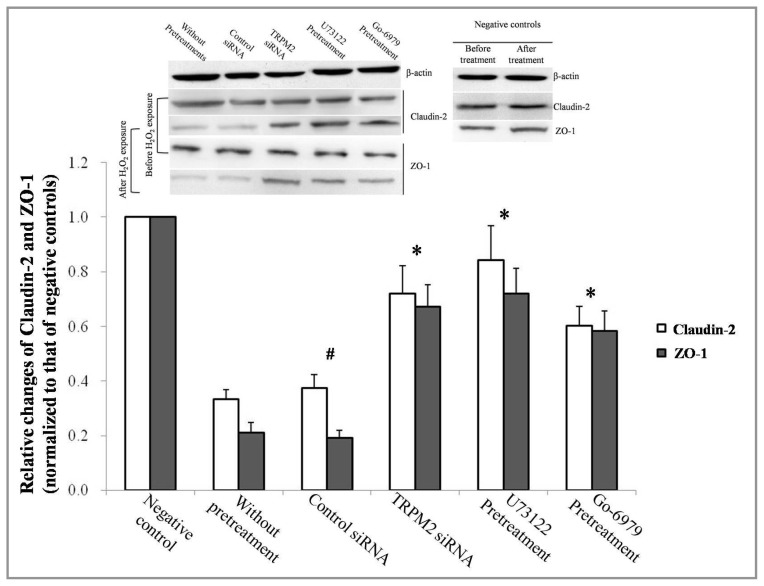
Expression levels of ZO-1 and claudin-2, the tight junction (TJ) protein family, were detected by western blot analysis. The results were normalized with respect to β-actin levels and adjusted to negative controls. 16HBE cells exposed to H_2_O_2_ free DMEM for 4 h were set as negative controls. (*n* = 6 for each condition), * *p* < 0.05 compared to the group without any pretreatments. ^#^*p* > 0.05 compared to the group without any pretreatments.

Because ZO-1 and claudin-2 maintain the integrity of the airway epithelium [12], Western blot analysis was used to detect both ZO-1 and claudin-2 levels following exposure to H_2_O_2_. As expected, ZO-1 and claudin-2 expression was attenuated after exposure to H_2_O_2_. However, less observable changes were detected if any key components of the TRPM2-PLCγ1-PKCα signaling cascade were blocked (Figure 3).

The epithelial tight junction barrier of the airway epithelium is stably maintained via the regulation of tight junction molecules expressed in epithelial cells. According to recent investigations, there is a strong correlation between oxidative stress and airway inflammation diseases [25,26]. ROS production has been correlated with tight junction injury and increased cell permeability [27]. Similar to the findings of Sun Y [7], our data demonstrated a reduction in ZO-1 and claudin-2 levels in 16HBE cells after H_2_O_2_ exposure. Furthermore, a decrease in TER of approximately 56% after exposure to H_2_O_2_ (Figure 2) strongly supports the participation of oxidative stress in the injury and hyperpermeability of airway epithelium.

The TRPM2 channel protein, an oxidation-sensitive TRP superfamily member, consists of six putative transmembrane domains with a pore formed by loops between the fifth and sixth segments [28]. Whole cell current measurements indicate that TRPM2 functions as a non-specific cation channel. While highly permeable to Na^+^ and K^+^, TRPM2 also exhibits considerable permeability to Ca^2+^ [16]. Oxidants, acquired externally or generated in the cytosol during oxidative stress, stimulate adenosine diphosphoribose (ADP-ribose) formation in the nucleus and mitochondria [29]. The free radical intermediates include superoxide anion (O^2−^), H_2_O_2_, nitric oxide (NO) and hydroxyl radical (OH), contribute to DNA oxidation and injury, which in turn initiates poly-ADP ribose polymerase (PARP)-mediated ADP-ribose generation [29]. PARP binds to single- and double-stranded DNA breaks and catalyzes the breakdown of NAD into nicotinamide and poly ADP-ribose. Free ADP-ribose is then produced from poly ADP-ribose degradation by poly ADP-ribose glycohydrolase (PARG) [30].

Following exposure to oxidative stress, the TRPM2 channel is induced at its *C*-terminus domain by the intracellular second messenger ADP-ribose, which is formed in the nucleus and mitochondria [29]. Activated TRPM2 enables Ca^2+^ influx and leads to a series of intracellular signals. Recent studies have implicated PKCα as a major component in increasing tight junction permeability [13]. Studies on the spatio-temporal location of PKCα have revealed PIP2-dependent translocation when activated by ATP [21]; additionally, the activation of PLCγ1 supports a related mechanism in the context of hypoxia and oxidative stress [31]. Therefore, we hypothesized that a PLC-related mechanism signals upstream of PKCα activation. To test our prediction, phosphorylation of PLCγ1 was detected by Western blot analysis. As predicted, H_2_O_2_ exposure significantly increased the phosphorylation of PLCγ1. Phosphorylation of PLCγ1 was dependent upon the function of TRPM2 during oxidative stress and subsequently, the activation of PKCα, which was responsible for the reduction of ZO-1 and claudin-2 in 16HBE cells. Inhibition of PLCγ1 phosphorylation by pretreatment with U73122, a PLC inhibitor, dramatically decreased PKCα activation, as well as the degradation of ZO-1 and claudin-2, during exposure to oxidative stress.

Present experiments closely linked extended activation of PKCα to impaired barrier function in both epithelial and endothelial cells [13,14]. As described by Song, J.C. *et al*., PMA-associated fall in TER only occurred after activation and translocation of PKCα from the basal cytoplasm to the apical zone [14]. Meanwhile, experiments on endothelial permeability indicated that impaired barrier function of endothelia induced by proinflammatory cytokines, such as IL-1β, could be prevented only by PKCα selective inhibitor Go6976, but not by other PKC selective inhibitors. In our investigation, the activity of PKCα was also suppressed by Go-6976, a PKCα specific inhibitor, in 16HBE cells before H_2_O_2_ exposure. Our findings are consistent with other studies and demonstrate the importance of PKCα in the degradation of TJs and epithelial barrier injury [13,14]. In summary, by using selective inhibitors and TRPM2 specific siRNA, we have been able to demonstrate the remarkable importance of TRPM2-PLCγ1-PKCα signaling chain in the hyperpermeability of airway epithelium induced by oxidative stress *in vitro* study.

## 3. Experimental Section

### 3.1. Materials

Human bronchial epithelial cells (16HBE) were purchased from American Type Culture Collection (Manassas, VA, USA). All of the antibodies used for Western blotting, including PLCγ1 antibody (Abcam, ab16955) (Cambridge, MA, USA), phosphorylated PLCγ1 Y783 antibody (Abcam, ab53125), PKCα antibody (Abcam, ab32376), ZO-1 antibody (Abcam, ab59720), claudin-2 antibody (Abcam, ab53032), β-actin antibody (Abcam, ab25894), Na^+^-K^+^ ATPase antibody (Abcam, ab76509), second antibody anti-mouse IgG (Abcam, ab6789) and anti-rabbit IgG (Abcam, ab97200) were purchased from Abcam (Cambridge, MA, USA). The transfection reagent FuGENE HD was acquired from Roche (Basel, Switzerland). Dulbecco’s Modified Eagle Medium (DMEM) and fetal bovine serum (FBS) were purchased from Gibco (Carlsbad, CA, USA).

### 3.2. Cell Culture and Treatment

The 16HBE cells were propagated in DMEM supplemented with 10% fetal bovine serum (FBS), 100 U/mL penicillin and 100 μg/mL streptomycin in a 37 °C, 5% CO_2_ incubator. Before treatment, 16HBE cells were plated in 6 × 60 mm culture dishes at a density of approximately 2 × 10^6^/mL and cultured in a 37 °C, 5% CO_2_ incubator to allow the cells to attach.

### 3.3. Small Interfering RNA Transfection

TRPM2-specific small interfering RNA with the vector pGCsilencerH1/hygro was synthesized and packaged by GeneChem (Shanghai, China). As a negative control, a base sequence containing a similar GC content was inserted into the vector. Before transfection, 16HBE cells in the exponential growth phase were plated at a density of approximately 2 × 10^6^/mL and incubated in culture dishes for 12 h. After washing with PBS 3 times to avoid interference by antibiotics and serum, the 16HBE cells were transfected using FuGENE HD with either TRPM2 siRNA or the negative control vector, according to the manufacturer’s recommendations. The siRNA concentrations were based on dose-response studies (data not shown).

### 3.4. H_2_O_2_ Exposure

16HBE cells incubated in the culture dishes were washed 3 times with chilled PBS and 30% (*v*/*v*) hydrogen peroxide solution was diluted in DMEM. In the preliminary experiment, different concentrations of H_2_O_2_ (0, 0.5 and 1 mM) and different exposure times (0, 2, 4 and 8 h) were tested with 16HBE cells. Cell viability was evaluated by a Typan staining assay. For optimal cell viability and maximum exposure, 0.5 mM H_2_O_2_ for 4 h were selected as the ideal exposure condition (Supplementary Figure S1).

### 3.5. Epithelial Barrier Function of 16HBE Cells

Epithelial barrier function was estimated by transepithelial electrical resistance (TER). 16HBE cells were seeded into transwell inserts at a density of 5 × 10^5^/well. Cells were cultured for the formation of intercellular adhesion. Cell layer TER was evaluated using the Millicell-ERS system (Millipore Co., Bedford, MA, USA) before and after H_2_O_2_ exposure. The TER values (Ohm × cm^2^) were calculated using the following equation: (TER sample-TER blank) × surface area.

### 3.6. Western Blot

The total membrane and cytosol proteins were extracted by following the protocol of the Membrane and Cytosol Protein Extraction Kit (Beyotime, P0033, Shanghai, China). Proteins from cell extraction were separated by SDS-PAGE and transferred to PVDF membranes. The PVDF membranes were blocked with 5% skim milk and incubated with primary antibodies: PLCγ1 at 1:2000 dilution (Abcam, ab16955); phosphorylated PLCγ1 at 1:300 dilution (Abcam, ab53125); ZO-1 at 1:100 dilution (Abcam, ab59720); or claudin-2 at 1:1,000 dilution (Abcam, ab53032) overnight. The PVDF membranes were washed 3 times with TBST and incubated with the corresponding secondary antibodies: anti-mouse IgG (Abcam, ab6789) or anti-rabbit IgG (Abcam, ab97200). Protein bands were visualized by enhanced chemiluminescence, following the manufacturer’s instruction (Beyotime ECL Plus, Shanghai, China). The intensity of each band was measured by a Fluor-S MultiImager and Quantity-One software (Bio-Rad, Hercules, CA, USA). Protein expression levels were normalized to those of β-actin.

### 3.7. PLCγ1 and PKCα Activity Assay

PLCγ1 could be phosphorylated at Tyr771, 783 and 1245. However, phosphorylation by Syk at Tyr783 activated the enzymatic activity of PLCγ1 [22,23]. Therefore, the activity of PLCγ1 was assessed by examining the phosphorylation of PLCγ1 at tyrosine 783 using Western blot analysis. To assess PKCα activity, 16HBE cells were gently rinsed 3 times in chilled PBS, followed by the addition of 250 μL of lysis buffer (containing 35 mM Tris-HCl, 0.4 mM EGTA, 10 mM MgCl_2_, 0.01% Triton X-100, 1 mM PMSF and 1 mM protease inhibitor) (Beyotime, China). After supercentrifugation, cytosolic fractions contained soluble proteins and unactivated PKC, while particulate fractions contained nonsoluble material (cell membranes, nuclear particles and cytoskeletal elements), as well as all translocated and activated PKC. The variation in the activity of PKCα was estimated by the differential quantity of particulate fractions and assessed by Western blot analysis (same as the “Western Blot” Section, mentioned above).

### 3.8. Statistical Analysis

Data were reported as x ± S.D. and analyzed by one-way ANOVA with SNK-q test for the comparison between each group. Statistical significance is indicated where *p* < 0.05.

## 4. Conclusions

Taken together, our study demonstrates that oxidative stress opens the TRPM2 ion channel, which enables Ca^2+^ influx. Subsequently, the phosphorylation of PLCγ1 is responsible for the activation and translocation of PKCα in 16HBE cells. The activation of PKCα results in reduction of ZO-1 and claudin-2, opening the TJs in the airway epithelium. Furthermore, 16HBE cells pretreated with a PLC inhibitor, a PKCα inhibitor or subjected to TRPM2 silencing exhibited increased resistance to hyperpermeability induced by H_2_O_2_. We speculate that oxidative stress opens airway TJs through a TRPM2-PLCγ1-PKCα signaling pathway.

## Supplemental Information



## Figures and Tables

**Table 1:** Transepithelial electrical resistance (TER) measurements of each group.

	TER values before treatments (Ω·cm^2^)	TER values after treatments (Ω·cm^2^)
Negative control	351.41 ± 30.91	379.39 ± 41.11 *
H_2_O_2_ exposure	360.83 ± 48.94 #	166.04 ± 34.31
Control siRNA	345.41 ± 26.92 #	148.78 ± 29.19
TRPM2 siRNA	376.67 ± 56.54 #	273.40 ± 44.80 *
U73122 pretreatment	349.05 ± 49.71 #	271.83 ± 38.50 *
Go-6976 pretreatment	364.42 ± 56.74 #	296.44 ± 29.99 *

In experimental group, 5 μM Go-6976 and 400 ng/mL U73122 were chosen as the inhibitor of PKCα and PLCγ, respectively. Before exposure to H_2_O_2_, TER values of each experimental group were recorded as the initial TER values. Followed by the exposure of 0.5 mM H_2_O_2_ for 4 h, as experimental section mentioned, the TER values of each experimental group were recorded again. However, in the negative control, 16HBE cells were exposed to H_2_O_2_ free DMEM for 4 h. (*n* = 6 for each condition).

* *p* < 0.05 compared to H_2_O_2_ exposure group,

# *p* > 0.05 compared to negative controls.
