# Supplementary material for: Oxidative Stress Mediates the Disruption of Airway Epithelial Tight Junctions through a TRPM2-PLCγ1-PKCα Signaling Pathway
Source: Int J Mol Sci. 2013 Apr 29;14(5):9475–86. doi: 10.3390/ijms14059475 (PMC3676794; doi:10.3390/ijms14059475)
Supplement: Supplementary file 1 [file ijms-14-09475-s001.pdf]

Supplemental Information

Figure S1. Trypan staining assay for the cell viability after H_2O_2 exposure. Cell viability was evaluated by the proportion of Trypan blue cells in total cells (n = 6). $^{\#}$ $p > 0.05$ compared to the treatment of H_2O_2 free cultrure medium for 4 h. * $p < 0.05$ compared to the treatment of H_2O_2 free cultrure medium for 4 h. ▲ $p < 0.05$ compared to the treatment of H_2O_2 free cultrure medium for 2 h. ▼ $p < 0.05$ compared to the treatment of H_2O_2 free cultrure medium for 8 h.

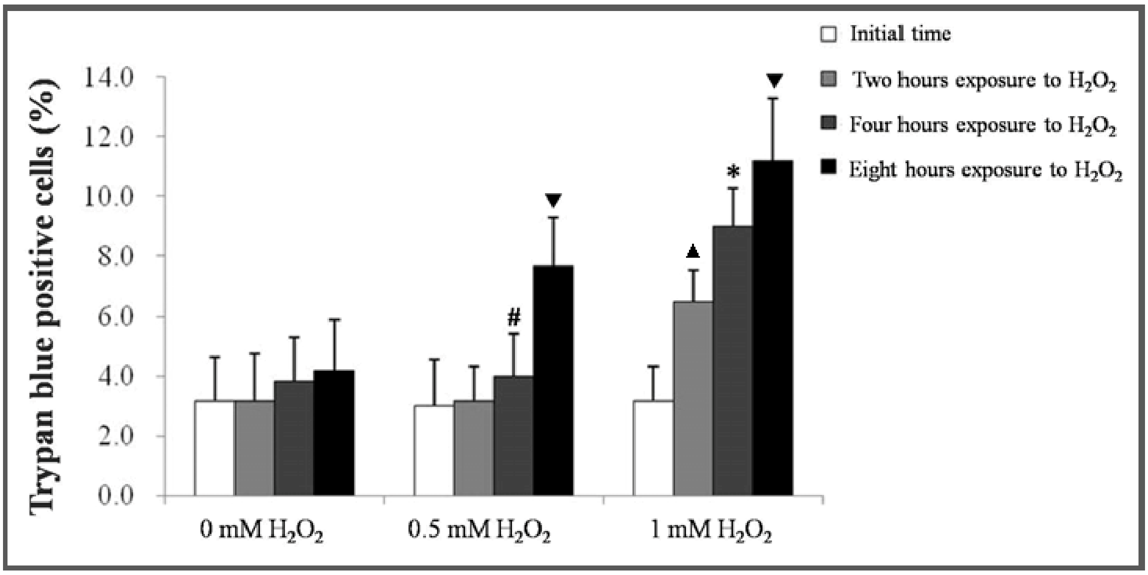

© 2013 by the authors; licensee MDPI, Basel, Switzerland. This article is an open access article distributed under the terms and conditions of the Creative Commons Attribution license (http://creativecommons.org/licenses/by/3.0/).
